# Ecological Survey of the Peridomestic Sand Flies of an Endemic Focus of Zoonotic Cutaneous Leishmaniasis in the South-East of Morocco

**DOI:** 10.1155/2022/5098005

**Published:** 2022-11-10

**Authors:** Zalalham Al-Koleeby, Ahmed El Aboudi, Wim Van Bortel, Kristien Cloots, Raja Benkirane, Chafika Faraj, Fatima Zahra Talbi

**Affiliations:** ^1^Laboratory of Medical Entomology, National Institute of Hygiene, 27 Avenue Ibn Battuta, Agdal, Rabat 11400, Morocco; ^2^Plant and Microbial Biotechnology, Biodiversity and the Environment, Faculty of Science, Agdal University, Rabat, Morocco; ^3^Unit of Entomology, Department of Biomedical Sciences, Institute of Tropical Medicine, Nationalestraat 155, Antwerp 2000, Belgium; ^4^Department of Public Health, Institute of Tropical Medicine, Antwerp, Belgium; ^5^National School of Public Health, Rue Lamfadel Cherkaoui, Madinat Al Irfane, Rabat BP-6329, Morocco; ^6^Hassan First University of Settat, Faculty of Sciences and Technologies, Laboratory of Biochemistry, Neurosciences, Natural Resources and Environment, P.O. Box 577, Settat 26000, Morocco

## Abstract

Leishmaniasis is a parasitosis caused by parasites of the genus *Leishmania* and is transmitted by Phlebotominae sand flies. An entomological survey was carried out in different localities of Zagora Province. Our work allowed us to establish an inventory of sand flies to study potential vectors of leishmaniasis and to compare the composition and the specific abundance of different endemic stations. The sand flies were collected using CDC miniature light traps during the month of July 2019 in the ten studied villages. The results indicate the presence of thirteen species, belonging to the genera *Phlebotomus* and *Sergentomyia*. *Phlebotomus papatasi* was the predominant species (46.65%) followed by *Ph*. *alexandri* (17%), *Ph*. *longicuspis* (11.55%), *Ph*. *bergeroti* (1.53%) and *Ph*. *sergenti* (1.27%). *Phlebotomus kazeruni* (0.03%) was rare, and only one female was captured in Ifred. *Sergentomyia schwetzi* (8.69%) was the most prevalent species in the *Sergentomyia* genus followed closely by *Se*. *fallax* (6.84%). *Sergentomyia africana* was present with a proportion of (3.86%) followed by *Se*. *clydei* (1.96%). *Sergentomyia dreifussi* (0.46%), *Se*. *antennata* (0.08%), and *Se*. *minuta* (0.08%) were very limited. *Phlebotomus papatasi*, *Ph*. *alexandri*, *Ph*. *bergeroti*, *Ph*. *longicuspis*, *Ph*. *sergenti*, *Se*. *schwetzi*, *Se*. *clydei*, and *Se*. *fallax* are constant species, being present at least in 50% of the stations (occurrence> 50%). Common species (25%–49%) were *Se*. *minuta* and *Se*. *africana* and rare species were *Ph*. *kazeruni* and *Se*. *antennata* with a very limited distribution (occurrence <12%). The greatest species richness was found in Ksar Mougni and Ifred with the occurrence of 11 species, but overall, it was high (>9 species) in most of the villages. The Shannon–Wiener index was high (*H*′ > 1) in eight localities (Ksar Mougni, Tassaouante, Bleida, ZaouiteLeftah, Ifred, Timarighine, Ait Oulahyane, and Ait Ali Ouhassou). The high value of this index is in favor of the ZaouiteLeftah locality (Shannon–Wiener index = 1.679) which is explained by the presence of a stand dominated by *Ph*. *papatasi*. In order to avoid exposure to infections, a good epidemiological surveillance and vector with rodent control measures must be well maintained. Awareness campaigns are also required and must be conducted for better knowledge of the disease.

## 1. Introduction

Sand flies are vectors of various pathogens responsible for human and/or animal diseases. Among these diseases, leishmaniasis is a public health problem in Morocco [1]. They are endemic in several regions of the country with two major clinical forms: cutaneous leishmaniasis (CL) and visceral leishmaniasis (VL). Cutaneous leishmaniasis caused by *Leishmania* (*L*.) *tropica*, endemic in the center and north of the country, and CL, caused by *L*. *major* and is endemic especially in the south and east of the country, are the most widespread diseases [2]. Visceral leishmaniasis caused by *L*. *infantum* occurs sporadically throughout the country but is particularly common in the north [3, 4]. The sand fly species responsible for the transmission of each of these forms are different; *Phlebotomus papatasi* is the vector of *L*. *major*; *Ph*. *sergenti* is responsible for the transmission of *L*. *tropica*, while *L*. *infantum*is is transmitted by three species *Ph*. *perniciosus*, *Ph*. *longicuspis* and *Ph*. *ariasi* [5]. Until now, 24 sand fly species have been identified in Morocco, of which 14 species belong to the *Phlebotomus* genus and 10 to the *Sergentomyia* genus [6]. The diversity of sand flies, their abundance, and distribution are influenced by several geographic and bioclimatic factors. Thus, entomological field investigations are of great importance for identifying these factors and their impact on each species and especially on the vector species. This study is considered as the first research on the diversity of sand flies in different biotopes of Zagora Province which is the most infected focus of zoonotic cutaneous leishmaniasis (ZCL) in Morocco [7]. It aims to compare the composition of sand flies species in ten localities and to identify the main high-risk biotopes due to the presence of vector species.

## 2. Material and Methods

### 2.1. Study Region

**Figure 1:**
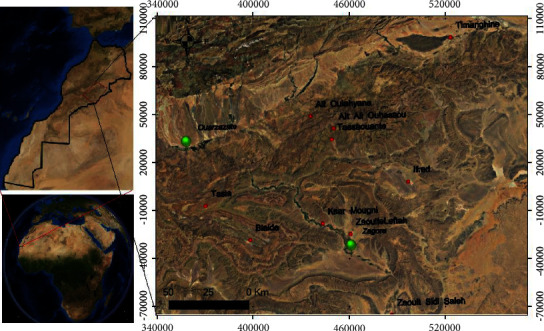
Study area and location of the ten study villages in Zagora Province.

The study was conducted in the province of Zagora (30°19′50″ N, 05°50′17” W) in the south-east of Morocco. The weather conditions are typical of the Saharan climate, i.e., hot in summer and quite cold in winter. The mean annual precipitation is 61 mm, and the mean annual temperature is 23°C. A variety of domestic animals that are potential sand fly hosts (sheep, goats, donkeys, chickens, rabbits, cats, and dogs) are present in this area. Ten villages were monitored for the sand fly presence (Figure 1).

### 2.2. Sand Flies Collections

The sand flies were collected using CDC miniature light traps (LT) during July 2019 in the ten study villages. July was chosen to conduct this survey since it corresponds to the period when sand flies reach their optimal abundance in Zagora Province [8, 9]. Five houses were randomly chosen in each village where two CDC traps were set for three consecutive nights from sunset to sunrise, i.e., one inside (bedrooms) and the other outside (courtyard). In the ten prospected localities, a total of 300 LT were set up during 30 nights of trapping. Captured specimens were transferred into tubes with 90% ethanol and were labeled. Prior to species identification, sand flies were cleared in the Marc André solution (chloral hydrate/acetic acid) [10]. Specimen identification was individually verified based on the morphology of the pharynges and/or the male genitalia or female spermathecae, as described by Faraj and Himmi [6]. In a second step, they were counted and separated by sex.

### 2.3. Data Analysis

The data were processed by ecological indices of composition. These were calculated as follows:

- Specific richness (*S*) is the number of species in a given area.
- Relative abundance (*p*_*i*_) was calculated as follows: Number of specimens of species (*x*)/Total number of specimens × 100.
- Shannon–Wiener index (*H′*) was used to calculate species diversity: *H*′=−∑_*i*=1_^*S*^*p*_*i*_*lnp*_*i*_, where *S* is the number of species and *p*_*i*_ is the number of specific species divided by the total number of collected sand flies in each biotope.
- Evenness (Equitability index): *E* = *H*′/ln (*S*), where H′ is the value of Shannon–Wiener Index, and *S* is the species richness
- Simpson's Index: *D* = Σni (ni − 1)/*N* (*N* − 1), where ni is the number of individuals of the given species, and *N* is the total number of individuals.
- Degree of presence: *C* = *n*/*N × *100, where *n* is the number of localities where the species was found, and *N* is the total number of study localities. According to this index, sand fly species were classified as constant species that were found in 50% or more of the study localities; common species were captured in 25%–49% of the study localities, accidental species were found in 12.5%–24% of the study localities, and very accidental species were captured in less than 12% of the study localities [11].
- The sex ratio (male/female) was also calculated for each species collected during this study.

We used descriptive statistics with graphics and tables to interpret results. We studied the distribution of sand flies species using Qgis 2.18 software by integrating data on leishmaniasis in the geographic information system.

## 3. Result

### 3.1. Diversity of Phlebotomine Species

During the trapping period, 3463 specimens were collected in the ten studied villages. Their identification revealed the presence of 13 species belonging to two genera: six *Phlebotomus* species (78%) and seven *Sergentomyia* species (22%). Among the *Phlebotomus* species, *Ph*. *papatasi* was the predominant species (46.65%) followed by *Ph*. *alexandri* (17%), *Ph*. *longicuspis* (11.55%), *Ph*. *bergeroti* (1.53%), and *Ph*. *sergenti* (1.27%). *Phlebotomus kazeruni*(0.03%) was rare, and only one female was captured in Ifred. *Se*. *schwetzi* (8.69%) was the most prevalent species in the *Sergentomyia* genus followed closely by *Se*. *fallax* (6.84%). *Sergentomyia africana* was present with a proportion of (3.86%) followed by *S*. *clydei* (1.96%). *Se*. *dreifussi* (0.46%), *Se*. *antennata* (0.08%), and *Se*. *minuta* (0.08%) were very limited (Table 2). The sex ratio for the most abundant species was 1.83. The species specific's sex ratio varied according to the species concerned (Table 2).

### 3.2. Spatial Distribution of Phlebotomine Species

**Figure 2:**
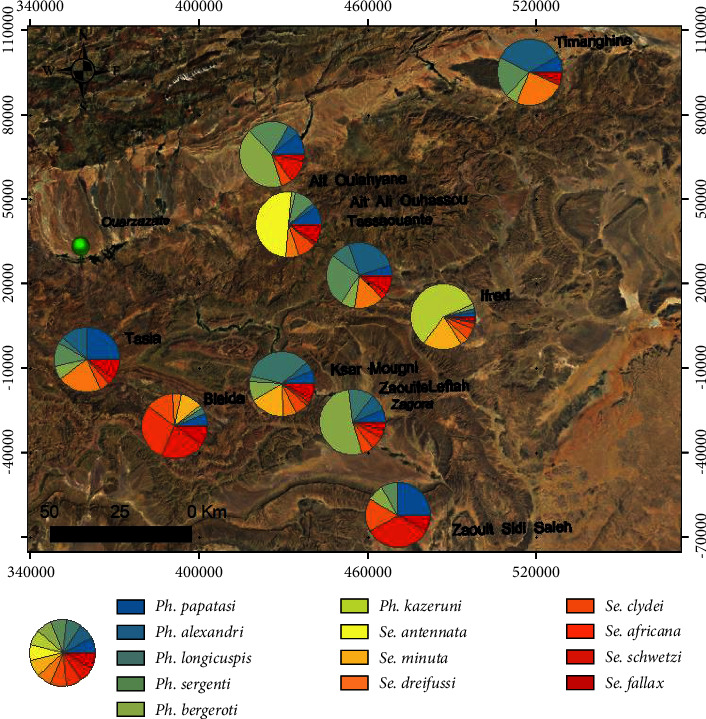
Spatial distribution of sand flies collected in Zagora Province.

**Figure 3:**
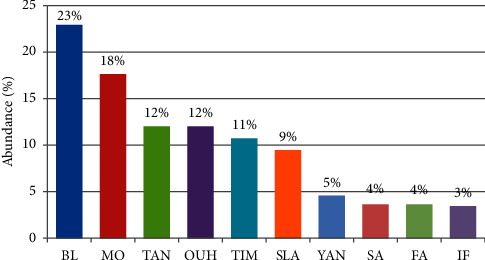
Abundance of sand flies in the ten villages studied

In terms of abundance, the highest number of sand flies was found at Bleida locality (BL) (23%), followed by KsarMogni (MO) locality (18%). Tassaouante (TAN) and Ait Ali Ouhassou (OUH) localities each accounted for 12% of the catches, and Timarighine (TIM) accounted for with 11%. Five localities accounted for less than 10% of the total catches, Tasla (SLA) (9%), Ait Oulahyane (YAN) (5%), Zaouit Sidi Saleh (SA) (4%), ZaouiteLeftah (FA) (4%) and Ifred (IF) (3%) (Figure 3). Concerning the distribution of sand fly species between villages, 38% of *Ph*. *papatasi* specimens were captured in the BL and OUH localities and 28% in MO and SLA localities. The remaining 34% were shared by the remaining six localities. The majority of *Ph*. *alexandri* specimens were collected at TIM locality (36%) then TAN (32%). *Phlebotomus longicuspis* was particularly trapped at MO locality (68%) (Figure 2). Species composition differed between villages. Overall, *Ph*. *papatasi*, *Ph*. *alexandri*, and *Ph*. *longicuspis* were collected from all villages. *Phlebotomus papatasi* was found to be predominant in 7/10 villages. *Phlebotomus alexandri* was the most prevalent species in two villages, Timarighine (57%) and Tassaouante (45%), followed by the two villages with a relative abundance of *Ph*. *papatasi* by about (33%) and (29%), respectively. *Phlebotomus longicuspis* predominated in only one village (KsarMougni (68%)), followed by *Ph*. *papatasi* (34%) (Figure 2).

### 3.3. Analysis of Sand Flies by Ecological Indices

The greatest species richness was found in MO and IF with the occurrence of 11 species, but overall, it was high in most of the villages. However, there were differences in species diversity, as indicated by the values of the Shannon–Wiener index (H′), evenness, and Simpson index among the villages (Table 1).

In order to characterize the population of sand flies captured in the different surveyed localities, fundamental ecological parameters were used: total richness (*S*), Shannon index (*H*′), and equitability index (*E*). A low value reflects a stand dominated by one species or a stand with a small number of species with a high representativeness. The Shannon–Wiener index is high (*H*′ > 1) at eight localities (MO, TAN, BL, FA, IF, TIM, YAN, and OUH). The high value of this index is in favor of the FA locality (Shannon–Wiener index = 1.679) which is explained by the presence of a stand dominated by *Ph*. *papatasi*.

By analyzing the distribution and frequency of species present in the surveyed sites, three groups of species can be distinguished: constant species, that are defined as being present in at least 50% of the stations (occurrence> 50%) were *Ph*. *papatasi*, *Ph*. *alexandri*, *Ph*. *bergeroti*, *Ph*. *longicuspis*, *Ph*. *sergenti*, *Se*. *schwetzi*, *Se*. *clydei,* and *Se*. *fallax*. Common species, present in 25%–49% of stations were *Se*. *minuta* and *Se*. *africana*, and rare species(occurrence <12%) were *Ph*. *kazeruni* and *Se*. *antennata* (Table 3).

## 4. Discussion

We conducted this entomological survey in the Province of Zagora, which is a pre-Saharan region in south-eastern Morocco and is considered as an endemic focus of ZCL, where the last outbreak was between 2008 and 2010 which included 4,437 cases [12]. In 2010, Zagora experienced an epidemic Zoonotic Cutaneous Leishmaniasis (ZCL) caused by *L*. *major* with 1134 cases. Even during the year 2017, a last epidemiological study reported the emergence of a new outbreak with a high incidence in the region [13]. This survey revealed the presence of 13 species of sand flies among the 23 species listed in Morocco (Faraj et Himmi, 2019). The two genus present in the country, *Phlebotomus* and *Sergentomyia,* are represented by, respectively, six and seven species.

*Phlebotomus papatasi* was the predominant species, and it was present in all the studied localities in large proportions. *Phlebotomus papatasi* and *Ph*. *bergeroti* have been recognized as sympatric in Morocco, Algeria, Egypt, Sudan, and Ethiopia with possibilities of crosses [14].*Ph*. *papatasi* species that was first described in Morocco in 2015 [15] is present throughout the country and is among the most common species [16]. Its density has been shown to increase with aridity [17], and it is present in all peridomestic biotopes, including houses, animal shelters, and caves [18], preferably between 400 and 800 m of altitude [19]. *Phlebotomus papatasi* is a confirmed vector of *L*. *major* in Morocco [20–22] and has always been found in the vicinity of ZCL foci in the arid region of the northern edge of the Sahara Desert [24]. In Morocco, the province of Zagora is considered as an endemic focus for *L*. *major*. The type of climate in this province characterized by periods of drought, intermittently interspersed with violent storms deeply mark the biological cycles. In this study area, several factors which have been assumed to be important in favor of phlebotomine proliferation were present: vegetation cover, soil type, abiotic factors, etc. as well as the biological development of the reservoirs (rodents) [21, 25, 26]. In addition to this, other risk factors could participate in ZCL epidemic, for example, dumping pits, open sewers, and cattle manure near houses. The rodent reservoir host incriminated in the transmission of ZCL due to *L*. *major* is *Meriones shawi* [27].

A study was conducted at Zagora Province, the study of seasonal dynamics of sand flies, especially at Mougni locality, noted a seasonal abundance of adult *Ph*. *papatasi* with a first peak in June, and then sand fly numbers decreased steadily in July–September as the climate became hotter, and then increased slightly to mark a second peak clearly less important than the first peak durinh late September–October [8]. In another locality Touna in the same province, *Ph*. *papatasi* showed a similar trend, but with only one peak in early July [8].

In Morocco, the period of activity of *Ph*. *pappatasi* was determined in Marrakech in the southwest of the country [28], in Tinghir adjacent to Zagora province [29] and in Zagora [8].

In the three studies, *Ph*. *papatasi* evolves in two generations with two peaks of abundance, the first in late spring and the second in early autumn.

*Phlebotomus alexandri* is considered a proven vector of kala-azar in China, where it was found infested at a rate of 2% in the Xiujiang-Uygan region [30, 31]. The anthropophilic behavior of *Ph*. *alexandri* implicates it as a potential vector of *L*. *tropica* [32–34]. In Iraq, *Ph*. *alexandri* is a proven vector of VL caused by *L*. *infantum* [35]. In Iran, 108 individual and pooled samples of *Ph*. *papatasi* and *Ph*. *alexandri* from Khuzestan Province showed infection with *L*. *major* and L. *infantum* [36]. *Phlebotomus alexandri* is the third most common species caught in Iran (accounting for more than 17%), and it is clearly anthropophilic and can be infected by *L*. *infantum*. Although other sand fly species have been found to be naturally infected with promastigotes, *Ph*. *alexandri* is the first to be proven to be infected with *L*. *infantum* [37]. *Phlebotomus alexandri* is a proven vector of *L*. *donovani* in China [37] and of the parasites, causing cutaneous leishmaniasis in Iran, Iraq, Algeria, Djibouti, Greece, Morocco, Tunisia, and Yemen [38]. No study has confirmed the vector role of this species in Zagora Province. In our study, from an entomological point of view, the locality Timarighine had the highest number of specimens (35.82%). In Iran, en se basant sur alpha biodiversité, the analysis of this parameter showed that agricultural ecosystem of Khorramshahr County had the highest diversity on *Ph*. *alexandri* and *Ph*. *papatasi*due to maximal richness and diversity and also relatively high evenness [39]. The factors for having high diversity of sand flies in the plain area studied may be due to higher annual precipitation, the related land use, and land cover. The changes on the composition of sand flies are perhaps due to human intervention in their natural habitats.

*Phlebotomus longicuspis* is native to the western part of the Mediterranean basin [5]. This species is considered to be widespread, preferentially in the subhumid to semiarid climatic stage. *Phlebotomus longicuspis* was the suspected vector of VL in Morocco [40]. In Zagora Province, this species represents only 11.55% of the total catch. Mougni locality has the highest abundance of *Ph*. *longicuspis* with 68%.

*Phlebotomus sergenti* is the main vector of *L*. *tropica*. Its role as a vector has been proven first in Saudi Arabia [41] and then in Morocco [42]. This role was also confirmed in Israel [43] and Ethiopia [44]. In Morocco, *Ph*. *sergenti* is known as a widespread species throughout the country and predominant in the arid and Saharan areas [45–47]. The vector is abundant in rural areas as well as in the habitats of the urban population [48]. In the present study, *Ph*. *sergenti* was present in seven localities, contributing to only 1.27% of the total sand fly catches. Tassaouante was the locality with the highest abundance of this species.

*Phlebotomus kazeruni* covers the sub-Saharan area. It is known in studies from Afghanistan [49], Iran [50], and Saudi Arabia [51]. Rioux et al. reported the presence of *Ph*. *kazeruni* in Southern Morocco [8]. In our study area, *Ph*. *kazeruni* is not very abundant. It represents only 0.03% of the species collected.

For *Sergentomyia* genus, the most dominant species is *Se*. *schwetzi* (8.69%), followed closely by *Se*. *fallax* (6.84%). *Se*. *africana*, *Se*. *clydei*, *Se*. *dreifussi*, *Se*. *antennata*, and *Se*. *minuta* were very limited. The species of the genus *Sergentomyia* have no impact on human health. They are herpetophilic species. They have been implicated in the transmission of Sauroleishmania and Trypanosomatids seen among reptiles in the old world [52]. Although some species feed on humans, they do not cause any epidemiological risk to humans until proven otherwise [53].

According to the distribution of species in the different localities, we can distinguish three groups of species. *Ph*. *papatasi*, *Ph*. *alexandri*, *Ph*. *longicuspis*, *Ph*. *bergeroti*, *Ph*. *sergenti*, *Se*. *schwetzi*, *Se*. *fallax*, *Se*. *clydei*, and *Se*. *dreifussi* are qualified constant because they were found in more than 50% of the localities surveyed. While *Se*. *africana* and *Se*. *minuta* are considered constant because they represent a frequency of occurrence between 25% and 49%. Then, the accidental species which are *Ph*. *kazeruni* and *Se*. *antennata* have a frequency of occurrence between 10% and 25%. In general, the diversity index is an ecological index that reflects the way individuals are distributed among species at a given time. In general, a high index reflects a balanced and diverse community, in which species are numerous, with no one species being truly dominant [54]. In our study, Zaouite Leftah (FA) is the locality with the highest index (*H*′ = 1.679), where eight species were found. The equitability in this environment is 70%, and it appears to be the best organized and structured station.

In order to verify risk factors involved in the proliferation of sand flies and favoring the biological development of the parasite vector *Ph*. *papatasi*, additional ecological and environmental studies of the region should be developed.

## 5. Conclusion

This entomological study in the Province of Zagora, Morocco, revealed a specific richness of phlebotomines of the order of 13 species including 6 of the *Phlebotomus* genus. The predominance of *Ph*. *papatasi* constitutes the basis for this province to be an *L*. *major* endemic area. In ZCL endemic foci, good surveillance and vector and rodent control measures must be well maintained. In addition, awareness campaigns are required and must be conducted for better knowledge of the disease in order to avoid exposure to infections.

## Figures and Tables

**Table 1:** Species richness, Shannon–Wiener diversity index (H), evenness (E), Simpson's diversity index (D) in studied villages.

Localités	Species richness	Shannon-wiener	Evenness	Simpson
Ksar Mougni (MO)	11	1.397	0.582	0.673
Tasla (SLA)	9	0.926	0.386	0.422
Zaouit Sidi Saleh (SA)	8	0.900	0.375	0.439
Tassaouante (TAN)	9	1.477	0.616	0.702
Bleida (BL)	10	1.608	0.671	0.750
Zaouite Leftah(FA)	8	1.679	0.700	0.768
Ifred (IF)	11	1.554	0.648	0.672
Timarighine (TIM)	9	1.085	0.452	0.565
Ait Oulahyane (YAN)	7	1.234	0.515	0.573
Ait Ali Ouhassou (OUH)	10	1.017	0.424	0.438

**Table 2:** Number of specimens and relative abundance of sand fly species collected in the ten villages of Zagora Province.

Species	Male	Female	Total	Ratio sex	Relative abundance (%)
*Ph*. *papatasi*	1045	569	1614	1.83	46.65
*Ph*. *alexandri*	219	370	589	0.59	17
*Ph*. *longicuspis*	144	256	400	0.56	11.55
*Ph*. *sergenti*	26	18	44	1.44	1.27
*Ph*. *bergeroti*	19	34	53	0.56	1.53
*Ph*. *kazeruni*	0	1	1	—	0.03
*Se*. *clydei*	26	42	68	0.62	1.96
*Se*. *africana*	107	27	134	3.96	3.86
*Se*. *schwetzi*	267	34	301	7.85	8.69
*Se*. *fallax*	145	92	237	1.57	6.84
*Se*. *dreifussi*	0	16	16	—	0.46
*Se*. *minuta*	0	3	3	—	0.08
*Se*. *antennata*	0	3	3	—	0.08
Total	1998	1465	3463	1.36	100

**Table 3:** Degree of presence of sand flies in study villages.

Species	Occurrence frequency (%)	Occurrence class
*Ph*. *papatasi*	100	Constant
*Ph*. *alexandri*	100	
*Ph*. *bergeroti*	100	
*Se*. *clydei*	100	
*Se*. *schwetzi*	100	
*Se*. *fallax*	100	
*Ph*. *longicuspis*	90	
*Ph*. *sergenti*	70	
*Se*. *dreifussi*	70	

*Se*. *africana*	40	Common
*Se*. *minuta*	30	

*Ph*. *kazeruni*	10	Very accidental
*Se*. *Antennata*	10	

## Data Availability

All the data analyzed during the study are included with links in the paper.
